# The role of tissue microstructure and water exchange in biophysical modelling of diffusion in white matter

**DOI:** 10.1007/s10334-013-0371-x

**Published:** 2013-02-27

**Authors:** Markus Nilsson, Danielle van Westen, Freddy Ståhlberg, Pia C. Sundgren, Jimmy Lätt

**Affiliations:** 1 Department of Medical Radiation Physics, Lund University, Lund, Sweden; 2 LBIC, Lund University Bioimaging Center, Lund University, 221 85, Lund, Sweden; 3 Department of Diagnostic Radiology, Lund University, Lund, Sweden; 4 Center for Medical Imaging and Physiology, Skåne University Hospital, Lund, Sweden

**Keywords:** Diffusion weighted imaging, Microstructure, Exchange, Permeability, White matter

## Abstract

Biophysical models that describe the outcome of white matter diffusion MRI experiments have various degrees of complexity. While the simplest models assume equal-sized and parallel axons, more elaborate ones may include distributions of axon diameters and axonal orientation dispersions. These microstructural features can be inferred from diffusion-weighted signal attenuation curves by solving an inverse problem, validated in several Monte Carlo simulation studies. Model development has been paralleled by microscopy studies of the microstructure of excised and fixed nerves, confirming that axon diameter estimates from diffusion measurements agree with those from microscopy. However, results obtained in vivo are less conclusive. For example, the amount of slowly diffusing water is lower than expected, and the diffusion-encoded signal is apparently insensitive to diffusion time variations, contrary to what may be expected. Recent understandings of the resolution limit in diffusion MRI, the rate of water exchange, and the presence of microscopic axonal undulation and axonal orientation dispersions may, however, explain such apparent contradictions. Knowledge of the effects of biophysical mechanisms on water diffusion in tissue can be used to predict the outcome of diffusion tensor imaging (DTI) and of diffusion kurtosis imaging (DKI) studies. Alterations of DTI or DKI parameters found in studies of pathologies such as ischemic stroke can thus be compared with those predicted by modelling. Observations in agreement with the predictions strengthen the credibility of biophysical models; those in disagreement could provide clues of how to improve them. DKI is particularly suited for this purpose; it is performed using higher *b*-values than DTI, and thus carries more information about the tissue microstructure. The purpose of this review is to provide an update on the current understanding of how various properties of the tissue microstructure and the rate of water exchange between microenvironments are reflected in diffusion MRI measurements. We focus on the use of biophysical models for extracting tissue-specific parameters from data obtained with single PGSE sequences on clinical MRI scanners, but results obtained with animal MRI scanners are also considered. While modelling of white matter is the central theme, experiments on model systems that highlight important aspects of the biophysical models are also reviewed.

## Introduction

The diffusion MRI experiment uses magnetic field gradients to label spins, as described pedagogically elsewhere [1, 2]. The most common design of the experiment is based on the pulsed-gradient spin-echo (PGSE) sequence, introduced by Stejskal and Tanner in 1965 [3]. Today, diffusion MRI is widely used in both neuroscience and for clinical applications, but already in 1965 Stejskal realised the technique’s potential for studying tissue: “living cells form a class of colloidal particles which should exhibit restricted diffusion of the substances confined within the cell walls” [4]. In addition to conventional experiments using a single pair of diffusion encoding gradients, the use of double gradient pairs for microstructural imaging has also been suggested [5, 6]. Such double pulsed-field gradient (d-PFG) experiments were later employed for investigations of microscopic anisotropy [7–10], estimation of compartment sizes [10, 11], and increasing the sensitivity to water exchange [12, 13]. Investigations using oscillating gradient waveforms represent another class of diffusion experiments, capable of exploring diffusion at very short diffusion times [14–17]. Non-conventional gradient waveforms have also been investigated [18].

Inferring information about the microstructure of tissue from the diffusion MRI experiment is an inverse problem, where models of the outcome of the experiment are fitted to the data acquired. The models describe the diffusion-weighted signal *S* for some experimental parameters, given the model parameters. Biophysical models of diffusion in white matter express *S* directly in terms of model parameters capturing tissue properties such as the axon diameter *d* and the fraction of water restricted in the intra-axonal space *f*_r_. Accurate quantification of the tissue properties requires the diffusion MRI experiment to be repeated several times with maximally varying experimental settings. This is typically achieved by the use of low- and high-diffusion sensitisation (high *b*-values), and long and short diffusion times [19]. Examples of biophysical models are the CHARMED and AxCaliber models [20, 21], and other similar models [19, 22, 23]. Phenomenological models, such as the diffusion tensor model used in diffusion tensor imaging (DTI) [24], kurtosis or generalized tensor model [25] used in diffusion kurtosis imaging (DKI) [26, 27], the stretched exponential model [28], and the ADC distribution model [29] also exist. Phenomenological models may show a high sensitivity for detecting alterations in the characteristics of the water diffusion, but do not assign the alterations to specific features of the tissue microstructure without further assumptions [30]. In addition to the phenomenological models, model-free approaches such as *q*-space analysis also exist, but they may be too sensitive to variations in experimental parameters to be useful in the analysis of data acquired with clinical MRI scanners [31, 32]. Given that the assumptions used when deriving biophysical models are valid, these models have the potential to increase the specificity of diffusion MRI by assigning alterations in the water diffusion characteristics to specific features of the tissue microstructure.

**Fig. 1:**
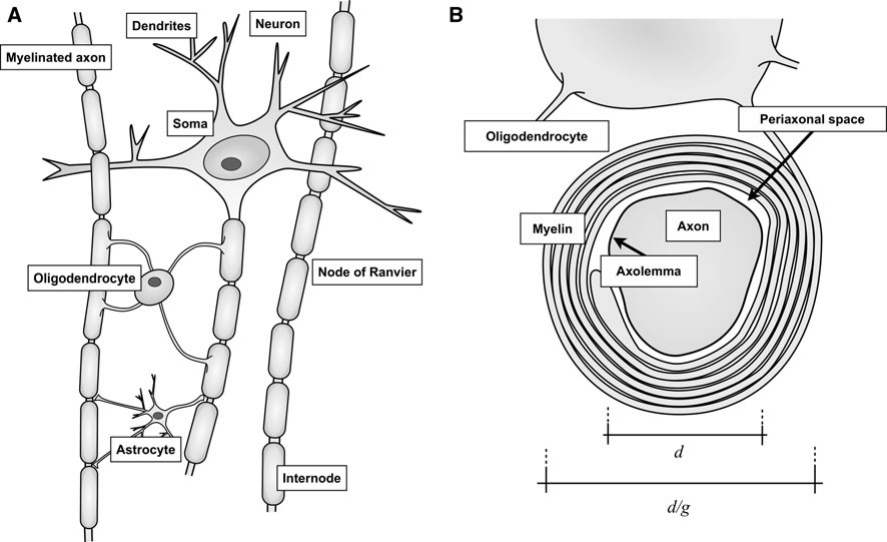
Drawing of the cell components in neural tissue (**a**) and myelin sheath structure (**b**), modified from Edgar and Griffiths [33]. **a** The cell body of the neuron, mainly found in *grey* matter, is also called the soma, from which several short dendrites and a one long axon extend. Some axons are encapsulated by myelin sheaths, which wrap around the axon like a balloon around a stick. The sheaths are extensions of oligodendrocytes. These generally form myelin sheaths around several axons. Narrow regions that are called nodes of Ranvier separate the sheaths. At these nodes, the axon membrane is exposed to the extracellular space. The segment between two nodes is called an internode. White matter also contains star-shaped glial cells called astrocytes. These support axons, for instance by regulating the extracellular ion concentration. **b** The ratio between the axon diameter *d* and the total axon diameter including the myelin is given by the *g* ratio. A small space exists between the axolemma and the inner part of the myelin sheath, called the periaxonal space, which is approximately 15 nm wide and filled with extracellular fluid

Modelling of water diffusion in tissue requires knowledge of the various microscopic environments in which the water molecules are located (Fig. 1a), since the properties of those environments impact the diffusion-encoded MRI signal. The glial cells are the most numerous cell type in the human brain, but these cells are small and thus constitute less than half of the human brain volume [33, 34]. For modelling of white matter diffusion, the most important structure is instead the axon [35]. The majority of vertebrate axons with diameters above 0.2 μm are myelinated, i.e., surrounded by a fatty sheath, although unmyelinated axons may have diameters of up to 1.8 μm [33]. In the human corpus callosum and other structures in the brain, most myelinated axons have diameters below 3 μm [36, 37]. Axons in the spinal cord and in peripheral nerves are generally larger than in the brain. For example, axons are between 3 and 9 μm wide in the mouse sciatic nerve [38], compared to 0.2 and 1.0 μm in the mouse corpus callosum [39]. Axons are also characterized by the ratio between their diameter and the outer diameter of the myelin sheath (*g*-ratio; Fig. 1b). The value of *g* is normally in the range 0.5–0.9, but varies as a function of age [33, 40, 41]. A value of exp(−½) ≈ 0.6 is optimal from a electrical conduction perspective [42]. Another important structural feature of axons are the so-called nodes of Ranvier, at which the axonal membrane (axolemma) is exposed to the extracellular space at gaps that are 0.8–1.1 μm wide (Fig. 1) [43]. The distance between the nodes (*L*) is between 100 μm and 2 mm, and increases with the axon diameter. Functionally, myelination, increased diameters and longer internode distances all contribute to increased signal transmission velocities in the axons [41, 42, 44], at the expense of the amount of energy required per transmission [45]. Finally, some axons display a wave-like undulating course, which allow nerves to stretch during motion, such as eye movement and locomotion, without being damaged [46]. Axonal undulation is found generally in extra-cranial white matter, but is also present intracranially, for example, in the optic nerve [47, 48].

Water-channel proteins, so called aquaporins (AQP), represent another factor that may influence water diffusion in brain tissue [49]. These proteins are embedded in the cell membranes, increasing their permeability to water. The function of AQP in the healthy brain is only partially understood [50], but the channels are known to control water movement into and out of the brain in cells located at the border between brain parenchyma and major fluid compartments. They also facilitate astrocyte migration and alter neuronal activity. The expression of AQP can be altered in disease, for example, in brain oedema where the astrocytic AQP expression is upregulated. Tumours that upregulate AQP expression may also be more aggressive and it has been proposed that AQP inhibitors may slow tumour growth [50]. Aquaporins are thus attractive targets for the development of novel drug therapies [51]. Methods capable of detecting and quantifying alterations of the membrane permeability may thus find clinical use.

Understandably, neural tissue is more complex than what can be captured in relatively simple biophysical models. Estimates of biophysical model parameters should thus be compared to estimates acquired using gold-standard techniques. Obtaining reliable information regarding the three-dimensional structure of tissue and the membrane permeability in live tissue is difficult, however. Simulations and numerical methods provide an alternative for investigation of model performance in well-controlled conditions. Such understanding improves the interpretation of experiments performed in vivo or in excised nerves and cell suspensions. The purpose of this review is to provide an overview of the various components used to build biophysical models of diffusion in white matter, and to review their applicability based on simulation studies. Agreement and disagreement between model predictions and results obtained in model systems such as excised nerves and cell suspensions are also discussed. Finally, the implications of the topics discussed are considered for in vivo measurements and the clinically relevant application of ischaemic stroke.

## Model construction and simulation-based validation

The goal of this section is to describe models that predict the diffusion-encoded signal in white matter. We start from the very minimal model of diffusion in white matter, and gradually extend the model to include effects of variable axon diameter, axon diameter distribution, orientation dispersion and compartmental exchange. The biological rationale for each extension is provided, along with results from simulation studies that characterize the accuracy and precision in estimates obtained with the models.

The three experimental parameters that control the diffusion weighting in a PGSE experiment are the duration and time between the onset of the diffusion-encoding gradients, denoted *δ* and Δ, respectively, and the magnetic field gradient **g**. Together, these parameters define the wave-vector **q** according to **q** = (*γ*/2*π*)*δ* **g**, where *γ* is the gyromagnetic ratio. The diffusion-sensitisation factor *b* is given by *b* = (2*πq*)^2^ *·t*_d_, where *q* = |**q**| and the diffusion time *t*_d_ is defined by *t*_d_ = Δ − *δ*/3, assuming that the rise times of the gradients are much shorter than *δ*. We will use the variables *b*, *t*_d_ and *δ* as the experimental parameters relevant for the model outcomes, although other triplets, such as *q*, Δ, and *δ*, would work equally well.

### The very minimal model

Biophysical modelling of diffusion in white matter start by describing the MR signal by two components, of which one has hindered diffusion (subscript h) and the other restricted diffusion (subscript r), according to [20]

1 $$ S = S_{0} \left( {f_{\text{h}} S_{\text{h}} + f_{\text{r}} S_{\text{r}} } \right), $$

where *f*_h_ and *f*_r_ = 1 − *f*_h_ are the signal fractions of the hindered and restricted components, respectively. Under the idealised conditions present in simulations, these components represent extracellular and intracellular water. In complex neural tissue, this assignment may only be conditionally valid, as will be discussed. Also note that the signal fractions denote the relative water populations after considering effects of potentially differing longitudinal and transversal relaxation rates in the components. The signal of *S*_h_ and *S*_r_ in Eq. 1 is given by

2 $$ S_{\text{h}} = \exp \left( {{ - }b\,D_{\text{h}} } \right)\,{\text{and}}\,S_{\text{r}} = \exp \left( {{ - }bD_{\text{r}} } \right). $$

This model thus contains four parameters : *S*_0_, *f*_r_, *D*_h_ and *D*_r_. Without further assumptions, this model is identical to the biexponential model [52, 53]. Note that *D*_h_ and *D*_r_ are not bulk diffusion coefficients, but rather apparent diffusion coefficients (ADCs) that are influenced by the experimental parameters and properties of the tissue.

To model the anisotropic diffusion in white matter [54], we assume that the diffusion coefficient in white matter is cylindrically symmetric along the main axis of the nerve [22], represented by the vector **u**. We may thus decompose *D*_h_ and *D*_r_ into axial and radial diffusivities, denoted AD_h_/RD_h_ for the hindered component and AD_r_/RD_r_ for the restricted component. The decomposition is identical for the hindered and restricted component, and given by [20]

3 $$D_{{{\text{h}}/{\text{r}}}} = \, ({\mathbf{n}} {\cdot}{\mathbf{u}})^{2} {\text{AD}}_{{{\text{h}}/{\text{r}}}} + \, (1 - ({\mathbf{n}} {\cdot}{\mathbf{u}})^{2} ){\text{RD}}_{{{\text{h}}/{\text{r}}}} ,$$

where **n** is the diffusion encoding direction and **u** is specified by polar and azimuthal angles *θ* and *ψ*. In order to specify the very minimal model of diffusion in white matter, we make two assumptions. First, we assume that the axial diffusivity is identical in both components (AD_h_ = AD_r_ = AD), and that it is independent of *δ* and *t*_d_. Secondly, we note that under experimental conditions with limited gradient amplitudes, RD_r_ ≈ 0 for small axon diameters [55]. Equation 1 now provides the MRI signal *S* using six model parameters: *S*_0_, *f*_r_, θ, *ψ*, AD and RD_h_. For experiments performed with diffusion encoding perpendicular to the nerve (**n** · **u** = 0), the model can be simplified so that it describe the radial signal attenuation curve RS using only three model parameters: *S*_0_, *f*_r_, and RD_h_, i.e. RS = *S*_0_(*f*_r_ + [1 − *f*_r_] exp[−*b* RD_h_]). In isotropic tissue, this model for *RS* also describes *S* in any direction.

This highly simplistic model of diffusion in white matter is based on the recognition that it is the organisation of cell membranes around axons that mainly determines the diffusivity in white matter [35]. Features of white matter that are less relevant to the model include, for example, the neurofilaments in the axonal cytoplasm [56]. Internal susceptibility-induced gradients are also negligible [57]. The very minimal model neglects water in glial cells, which is assumed to be either in fast exchange with the extracellular space and thus a part of the hindered fraction [49], or to represent a negligible fraction of the total MR signal. Despite its simplicity, the very minimal model provides valuable insights; for example, it predicts that RD obtained in DTI is sensitive to the axon density according to [55, 58]

4 $$ {\text{RD}} \approx \left( {1 - f_{\text{r}} } \right){\text{RD}}_{\text{h}} , $$

when assuming that the axon density correlates with *f*_r_. This relation is also valid to describe the mean diffusivity (MD) in isotropic tissue such as many tumours, which has led to the use of MD as a proxy for the cellularity of tumours [59].

### The compartment model and the resolution limit

The very minimal model can be expanded to include the axon diameter *d*, by modelling RD_r_ as a function of the axon diameter, *d*, and the intra-axonal diffusion coefficient, *D*_intra_, as well as the experimental parameters *δ* and *t*_d_. In the analysis of restricted diffusion, it is informative to define two dimension-less variables *α* and *β* according to

5 $$ \alpha = 4\delta D_{\text{intra}} /d^{2} ,\quad \beta = 4\Updelta D_{\text{intra}} /d^{2} .$$

The value of RD_r_ can now be calculated by using the approximation of a Gaussian phase distribution (GPD) [60–62], according to

6 $$ {\text{RD}}_{\text{r}} \left( {\alpha ,\beta } \right) = k^{2} \left( {\alpha ,\beta } \right)d^{2} /2t_{\text{d}} . $$

For diffusion restricted to a cylinder and with gradients applied perpendicular to the main axis of the cylinder, *k* ^2^(*α*, *β*) is given by [63, 64]

7 $$ k^{2} \left( {\alpha ,\beta } \right) = \sum\limits_{m = 1}^{\infty } {\frac{{2\alpha a_{m} - 2 + 2{\text{e}}^{{ - \alpha a_{m} }} + \left( {2 - {\text{e}}^{{\alpha a_{m} }} - {\text{e}}^{{ - \alpha a_{m} }} } \right){\text{e}}^{{ - \beta a_{m} }} }}{{\alpha^{2} a_{m}^{3} \left( {a_{m} - 1} \right)}}} $$

where *a*_*m*_ is defined by $J^{\prime}(a_{m}^{{1/2}} ) = 0$, so that (*a*_*m*_)^1/2^ are the roots of the derivative of the Bessel function of the first kind and order one. Other expressions are available for diffusion restricted by parallel planes or a sphere [64].

The variable *D*_intra_ in Eq. 5 is often assumed to be scalar (i.e., isotropic intra-axonal diffusion), with a value equal to AD or fixed to a value obtained from the literature. This model thereby describes RS using four model parameters: *S*_0_, *f*_r_, RD_h_, and *d*, and will here on be denoted as the compartment model. A similar model was called the minimal model of white matter diffusion by Zhang et al. [65]. The reason for *D*_intra_ not being included as a free model parameter here is that its value is difficult to measure directly, since RD_r_ only approaches *D*_intra_ when *t*_d_ → 0. However, RD_r_ << *D*_intra_ under most experimental conditions when performing diffusion MRI on neural tissue.

**Fig. 2:**
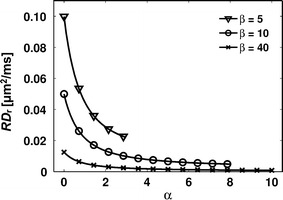
The value of RD_r_ quickly approaches zero as α increase. The graph illustrates Eq. 6 for various combinations of *α* and *β* in Eq. 5 for *d* = 4 μm, *D*_intra_ = 2 μm^2^/ms, and varying values of *δ* and ∆, so that α = 1 corresponds to *δ* = 2 ms. The maximum value of α is determined by *δ*_max_ = ∆ − *t*_rf_, here with *t*_rf_ = 4 ms. In practice, values of RD_r_ below approximately 0.02 μm^2^/ms may be difficult to distinguish from RD_r_ = 0

There is a lower limit that we call the resolution limit *d*_min_, below which the axon diameter is difficult to estimate accurately. The appearance of the resolution limit is evident in *q*-space analysis [31, 32, 66], but it appears also in model-based analysis. Alexander et al. [67] compared the accuracy of axon diameter estimates from acquisition protocols optimised for an animal and a clinical MRI scanner, featuring gradient systems with *g*_max_ = 140 and 60 mT/m, respectively. The study did not explicitly evaluate the value of *d*_min_, but it can be approximated from the results presented to 2.5 and 3.5 μm for the protocols optimised for the animal and human system, respectively. Nilsson et al. [68] similarly showed that axon diameter estimates are accurate only above 4–5 μm, based on results from Monte Carlo simulations performed for a protocol designed for a system with *g*_max_ = 100 mT/m. The inaccurate axon diameter estimates are caused by the quick approach of RD_r_ to zero as *d* decreases and *α* increases (Fig. 2). For example, RD_r_ ≈ 0.01 μm^2^/ms for *d* = 4 μm, *δ* = 10 ms, and Δ = 20 ms. In *q*-space analysis, the resolution limit is inversely proportional to the maximum *q*-value [66]. Reducing the resolution limit requires higher values of *g*_max_, which permits *q*_max_ to increase and *α* to decrease, which according to Lätt et al. [32] gives *d*_min_ ∝ *g*_max_^−1/3^. For model-based analysis, however, the resolution limit scales according to *d*_min_ ∝ *g*_max_^−1/2^, i.e., model-based analysis put less strong requirements on scanner hardware than *q*-space analysis does, according to preliminary results by Nilsson and Alexander [69]. In addition to being dependent on *g*_max_, the resolution limit also depends on the noise level. For example, Alexander showed that two systems with diameters of 2 and 4 μm became inseparable in terms of the estimated values of *d* when the signal-to-noise ratio (SNR) was reduced from 50 to 20 [19].

The compartment model relies on a few assumptions. First, it assumes that axons are well modelled by impermeable, parallel and equal-sized cylinders. This assumption can be relaxed, as discussed below. Secondly, it assumes that an inaccurate prior value of *D*_intra_ does not hamper the accuracy of other model parameters. To our knowledge, this assumption has not been investigated in detail. Thirdly, it assumes that RD_h_ is independent of *δ* and *t*_d_, which is probably an unproblematic assumption. Fourthly, it assumes that the GPD approximation describes RS_r_ sufficiently well. This assumption is valid for most experimental conditions [70], but not for *α* << 1 and *β* >> 1, since the signal curve then takes the shape of a diffraction pattern [71–73]. The amplitude of the highest diffraction peak is, however, less than 5 % of *S*_0_, although it may increase, for example, in the presence of a surface relaxation sink which enhance the relaxation rate close to the membrane [74]. Nevertheless, the GPD approximation is generally valid until less than 10 % of *S*_0_ remains, as shown both by simulations and experiments [68, 75]. Another condition that invalidates the GPD approximation is when *β* << 1. This condition may result in apparently biexponential signal-versus-*b* curves from a single compartment [76]. In the context of diffusion MRI using clinical MRI scanners, however, this condition is of little concern for most protocols since *β* << 1 only for *d* greater than 20 μm.

### Modelling of axon diameter distributions

Nerves are typically composed of axons of varying diameters, which can be incorporated in the model of *RS*_r_ according to

8 $$ {\text{RS}}_{\text{r}} = \int {\rho \left( {d^{\prime } |d,\sigma_{\text{d}} } \right)\exp \left( { - b\,{\text{RD}}_{\text{r}} \left( {d^{\prime } |\delta ,t_{\text{d}} } \right)} \right){\text{d}}d^{\prime } } , $$

where *ρ*(*d*′|*d*, *σ*_d_) is the volume-weighted axon diameter distribution with mean *d* and standard deviation *σ*_d_. This model will be referred to as the diameter distribution model, but it has also been described as the AxCaliber model [21]. In that model, the axon diameter distribution is modelled by a gamma distribution, with shape and scale parameters given by (*d*/*σ*_d_)^2^ and *σ*_d_^2^/*d*.

**Fig. 3:**
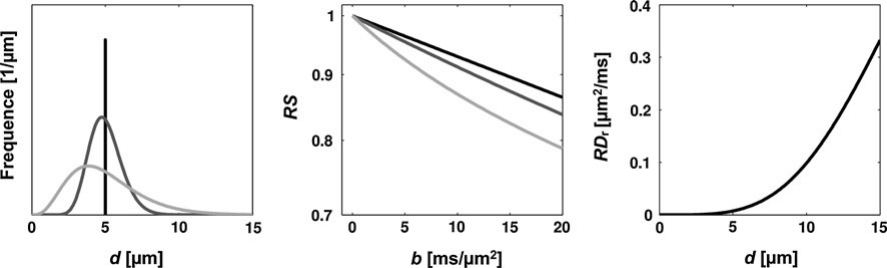
*Left*: Three gamma distributions of axon diameters, all with average diameters of 5 μm, but with *σ*_d_ = 0, 1.1 and 2.4 μm. *Middle*: corresponding RS_r_-versus-*b* curves from the three distributions, matched in *grey scale* with the distribution panel. *Right*: Values of RD_r_ used to generate the signal curves, versus *d*. The values were calculated from Eqs. 6 and 7, assuming a typical diffusion MRI protocol with *δ* = 20 ms and *t*_d_ = 18 ms. Note that all curves are approximately monoexponential up to the maximum *b*-value achievable with clinical MRI scanners (*b*_max_ ≈ 5 ms/μm^2^ for the given values of *δ* and *t*_d_, with *g*_max_ = 100 mT/m)

In the presence of a distribution of axon diameters, RS_r_ becomes apparently biexponential [77]. However, RS_r_ is approximately monoexponential under the experimental limitations imposed by the clinical MRI scanner, even for relatively large values of *σ*_d_ (Fig. 3). In the analysis of high *b*-value data acquired in vivo, Zhang et al. [65] showed in a conference abstract that a model assuming equal-sized axons produces higher estimates of the average axon diameter than a model assuming a diameter distribution. The source of this bias may be partly explained by Fig. 3, which shows that the slope of the signal-versus-*b* curve increases as *σ*_d_ increases, even as the average diameter is fixed. The estimated value of *f*_r_ is less influenced than the axon diameter by whether a single compartment size is assumed or a size distribution is incorporated in the model [78].

### Orientation dispersion and axonal undulation

Axons are normally modelled as being parallel, but this assumption may be invalid. Leergard et al. [79] obtained axonal orientation distributions by manually recording individual fibre orientations on myelin-stained histological sections. The full-width at half-maximum (FWHM) of the angular orientation distribution was 34° in the densely packed corpus callosum. Axonal undulation also induces axonal orientation dispersion [80].

Axonal orientation dispersion can be incorporated into the model of RS as described by Zhang et al. [81], but is here adapted to the form of Eq. 8, according to

9 $$ {\text{RS}}_{\text{r}} = \int {\rho \left( {{\mathbf{v}}|{\mathbf{u}},\kappa } \right)\rm{exp}\left( { - b\left( {{\mathbf{n}} \cdot {\mathbf{v}}} \right)^{2} {\text{RD}}_{\text{r}} - b\left( {1 - \left( {{\mathbf{n}} \cdot {\mathbf{v}}} \right)^{2} } \right){\text{AD}}_{\text{r}} } \right){\text{d}}{\mathbf{v}}} , $$

where *ρ*(**v**|**u**, *κ*) is the orientation distribution around the direction **u** with a dispersion factor *κ*. This model will be called the orientation dispersion model, described using five model parameters: *S*_0_, *f*_r_, RD_h_, *d*, and *κ*, assuming AD_r_ is fixed to some prior value.

The effect on RS_r_ of an orientation dispersion has been investigated experimentally and in simulations by Avram et al. [72]. The results showed that a wider orientation distribution led to faster signal attenuation at low *b*-values and less signal remaining at high *q*-values. Analysing such data with the compartment model would presumably result in higher values of RD_h_ and lower values of *f*_r_.

Zhang et al. [81] fitted the compartment model, which assumes parallel axons, to data simulated from the orientation dispersion model. This resulted in over- and underestimated values of *d* and *f*_r_, respectively, although these biases were almost recovered by instead fitting the orientation dispersion model to the simulated data. Effects of the resolution limit, however, prevented accurate estimation of *d* below approximately 4 μm. Drawing on the weak signal dependency for small axon diameters, Zhang et al. [82] refined the model to assume *d* = 0 μm, which allowed for improved estimation of *κ*. The resulting model, called neurite orientation dispersion and density imaging (NODDI), allows the orientation dispersion to be estimated in the human brain from data obtained in as little as 10 min.

**Fig. 4:**
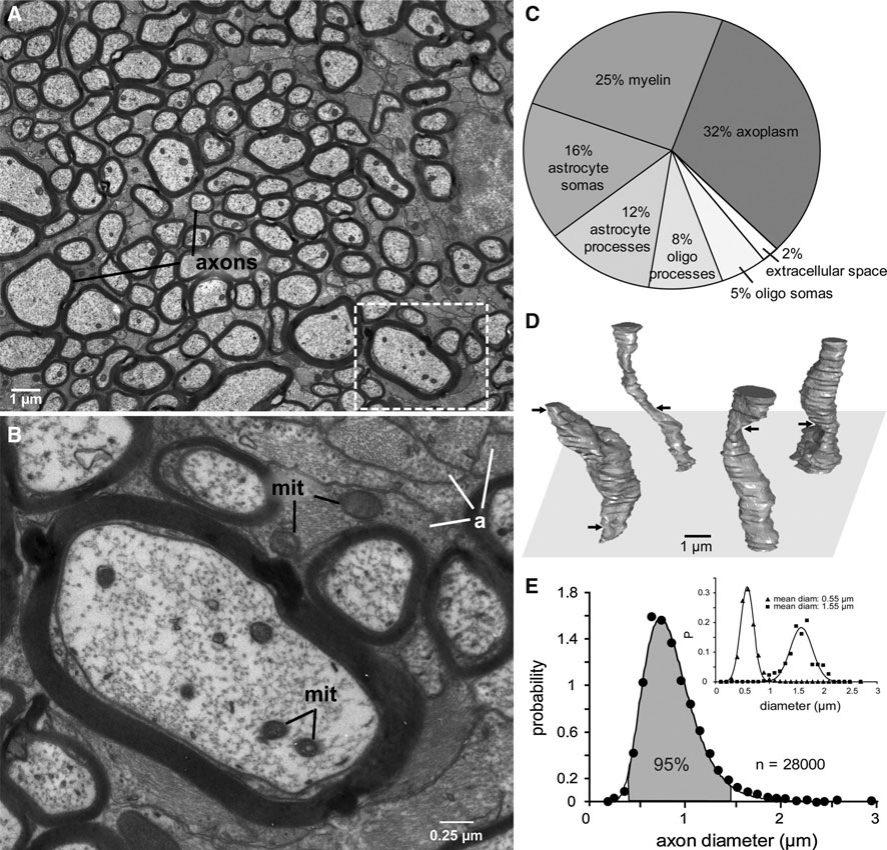
**a** Myelinated axons in the optic nerve vary in diameter by tenfold and are separated from each other by astrocyte processes (electron micrograph). **b** Higher magnification of the boxed region in A shows mitochondria (mit) in axons and astrocyte processes (**a**). **c** Allocation of space in the optic nerve. **d** Optic axons reconstructed from inner diameters over a length scale of 12 μm. The axons vary markedly in caliber (max/min = 2.0 ± 0.6 μm; *n* = 1,200). Arrows mark constrictions. None of these constrictions were nodes of Ranvier. **e** Distribution of diameters is skewed with thin axons predominating. *Solid line* is a lognormal fit. *Inset*: Distribution of diameters along the reconstructed segments for a subset of axons with mean diameter 0.55 μm (*n* = 1,100) and 1.55 μm (*n* = 500). Solid lines are Gaussian fits. Reproduced from Perge et al. [45] with permission from Journal of Neuroscience

Axons in extracranial white matter and in the optic nerve undulate, i.e., they follow approximately sinusoidal paths [83]. For the optic nerve, the non-straightness is easily appreciated from reconstructed 3D segments of axons (Fig. 4). Diffusion measurements performed in sinusoidally undulating axons yields results similar to those performed in the presence of orientation dispersion according to Monte Carlo simulations by Nilsson et al. [80], although there is a fundamental difference between orientation dispersion at the micro- and macroscopic levels. In axons that undulate with wavelengths of a few tens of microns, *d* is overestimated by an amount proportional to the undulation amplitude. This bias is probably not recoverable by improved modelling, since the water molecules have time to sample one or more complete undulations during the diffusion time, so that the effective restriction length is actually larger than the axon diameter. A similar argument can be applied for axons that vary in diameter, for example, those in the optical nerve which varies up to a factor of two in diameter over a distance of 12 μm [45]. In the case of undulation wavelengths of a hundred microns or more, the differently oriented segments of the axon may be regarded as non-exchanging [80], thereby meeting the assumptions in the orientation dispersion model. Stretching a nerve with undulating axons reduces the undulation amplitude, which would result in less of an overestimation of the axon diameter, and probably also in alterations of the diffusion characteristics measured by DTI [80].

### The two-compartment exchange model and membrane permeability

In the presence of exchange between two water components, the diffusion-weighted signal can be predicted by the Kärger equations [84, 85]. These equations are derived from the Bloch-Torrey equations [86], where the magnetisation **S** in the water components are related by rate equations d**S**/d*t* = **A**·**S**. The mixing matrix is given by **A** = −(2π*q*)^2^ **D** + **K**, so that the solution to the rate equations provides an expression for the total signal *S* according to [78]

10 $$ S\left( {q,t_{\text{d}} } \right) = {{S}}_{0} {\mathbf{1}}^{\text{T}} { \exp }\left( {-\left( { 2\pi q} \right)^{ 2} t_{\text{d}}  {\mathbf{D}} + t_{\text{d}} {\mathbf{K}}} \right) \cdot {\mathbf{f}}, $$

where *S*_0_ is the signal acquired without diffusion weighting, **1** is a column vector of ones. For the two-component system discussed previously, **D** = diag(*D*_h_, *D*_r_), i.e., the model assumes that the GPD approximation is valid in all compartments. Moreover, **f** = [*f*_h_, f_r_], and the exchange matrix **K** is given by

11 $$ {\mathbf{K}} = \left[ {\begin{array}{*{20}c} { - k_{{{\text{h}},{\text{r}}}} } & { + k_{{{\text{r}},{\text{h}}}} } \\ { + k_{{{\text{h}},{\text{r}}}} } & { - k_{{{\text{r}},{\text{h}}}} } \\ \end{array} } \right], $$

where conservation of mass gives *f*_h_ *k*_h,r_ = *f*_r_ *k*_r,h_ under the assumption that *f*_i_ represents the total mass of component *i*. For experiments performed using a double PGSE sequence instead of the conventional single PGSE sequence, the two-compartment model in Eq. 10 can be simplified to only include four model parameters in the so-called filtered exchange imaging (FEXI) experiment [87]. FEXI gives the apparent exchange rate (AXR), which is related to the exchange rate according to AXR = (*k*_r,h_·*f*_h_)^−1^. Details regarding that experiment are, however, outside the scope of this review.

For cells embedded in a homogeneous medium the outward exchange rate from the cells is given by

12 $$ K_{{{\text{r}},{\text{h}}}} = P_{\text{d}} \left( {A/V} \right)_{\text{r}} = {1/{\tau}} _{i} , $$

where *P*_d_ is the diffusional membrane water permeability, (*A*/*V*)_r_ is the surface-to-volume ratio of the cell, and *τ*_i_ is the mean residence time for a molecule in the cell, or the intracellular exchange time [13]. The diffusional water membrane permeability *P*_d_ is affected by the properties of the lipids in the membrane and by water-channel proteins embedded in the membrane [88, 89]. It generally increases smoothly with the temperature, although it may increase sharply at certain temperatures [13, 88]. Note the difference between the *osmotic* and *diffusional* permeability, where the former is generally larger than the latter and refers to the permeability measured in the presence of an osmotic pressure gradient over the membrane [90]. The diffusion NMR/MRI experiment measure the permeability under steady-state conditions, and thereby yields the diffusional permeability [91].

The model in Eq. 10 is here on called the two-compartment exchange model, and it describes RS using five parameters: *S*_0_, *f*_r_, RD_h_, *d*, and *τ*_i_. Special cases of this model allow *τ*_i_ to be inferred from constant-gradient experiments, in which *g* is fixed while *t*_d_ is varied [92, 93]. This approach provides accurate estimates of *τ*_i_, but for long diffusion times and values of *g*_max_ above those normally available with clinical MRI scanners. Instead of the approach used in constant-gradient experiments of only collecting limited data, a large set of experimental conditions with varying values of *δ*, *t*_d_ and *b* can be acquired. This allows the full two-compartment exchange model to be fitted to the data.

**Fig. 5:**
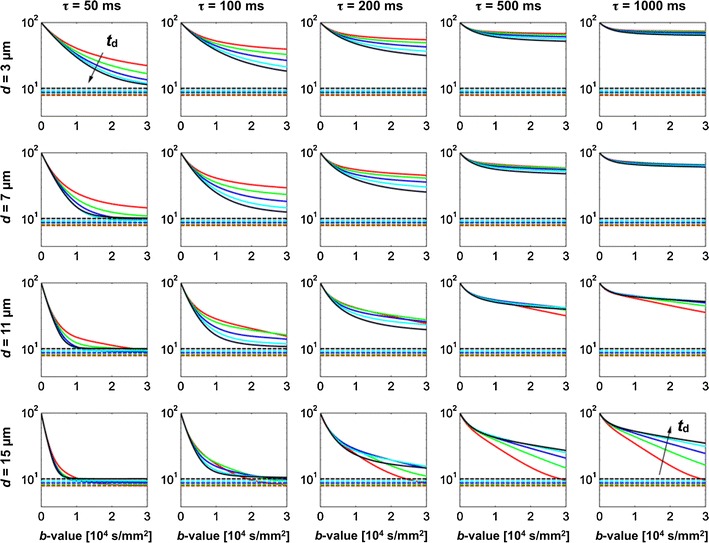
Signal curves simulated with *δ* = 50 ms and diffusion times from 64 to 256 ms, shown in *red* to *black*, order according to the *arrows*. *Columns* show varying exchange times, while rows show varying diameters. Note that the amplitude of the signal-versus-*b* curves increase at high *b*-values for prolonged *t*_d_ when effects of restricted diffusion dominate (*lower right*), while the opposite occurs when effects of exchange dominate (*upper left*). *Dashed lines* represent the magnified noise floor. Note the unit of *b*, where 10^4^ s/mm^2^ = 10^6^ s/cm^2^ = 10 ms/μm^2^. Reproduced from Nilsson et al. [23] with permission from Elsevier

Although the two-compartment exchange model is derived based on an assumption incompatible with the notion of restricted diffusion; that both components show Gaussian diffusion where the mean-squared distances increase linearly with time, it predicts the outcome of a single PGSE experiment well in most cases [68, 86]. For example, Nilsson et al. [23] evaluated the performance of the model using Monte Carlo simulations, for a protocol with *δ* = 50 ms, *t*_d_ = 64–256 ms, and *b*_max_ = 28 ms/μm^2^. The results showed that effects of both restricted diffusion and exchange can be observed for some microstructural configurations in signal-versus-*b* curves obtained using a clinical scanner (Fig. 5). Another study performed a similar evaluation using a protocol with *δ* = 30 ms, *t*_d_ = 30–60 ms, and *b*_max_ = 20 ms/μm^2^ [68]. These two studies showed that the two-compartment model generally provides accurate estimates of the values that were used in the simulation, except for *d* below the resolution limit. In addition, two other exceptions were found. First, the exchange time was accurately estimated only when being on the same order of magnitude as the maximal diffusion time employed in the measurements. For example, Nilsson et al. [68] showed that *τ*_i_ was accurately estimated for *τ*_i_ < 300 ms, compared to the maximal diffusion time of *t*_d_ = 60 ms. Second, fast exchange demand high *q* values in order to be observable according to the “shutter speed” analysis of Lee and Springer [94], and is accurately quantified only if the exchange is barrier limited [86].

**Fig. 6:**
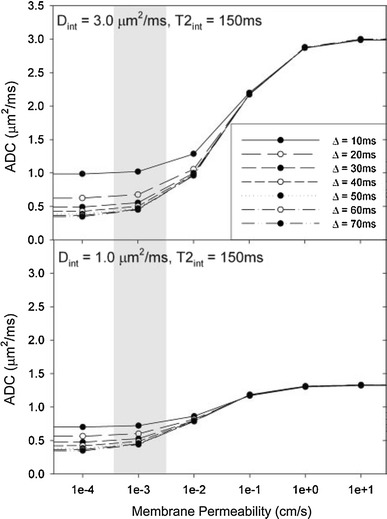
Variations in cell membrane permeability impact the ADC strongly for very high permeability values only. Calculated ADC values of water were plotted against the membrane permeability, with lines connecting simulations with identical diffusion times. *Top* and *bottom* panels depict simulation results with a combination of *D*_intra_ = 1.0 and 3.0 μm^2^/ms, respectively, with equal relaxivities in the two compartments of 150 ms. Shaded regions highlight physiologically relevant membrane permeability values in healthy cells. Reproduced from Harkins et al. [97], with permission from John Wiley and Sons

The concept of barrier limited exchange relates to an assumption in the Kärger equations; that the exchanging components are well mixed so that all particles have equal probabilities of switching components during *τ*_i_. This assumption is valid in compartmentalised systems only when *τ*_i_ >> *d* ^2^/2*D*_i_, i.e., barrier limited exchange as discussed by Fieremans et al. [86]. Violation of this condition leads to inaccurate parameter estimates. For example, Nilsson et al. [23] showed that the estimated values of *τ*_i_ and *f*_r_ became inaccurate for *d* > 8 μm. Another study by Nilsson et al. [68] similarly found that *f*_r_ was underestimated for large values of *d* and low values of *τ*_i_, but showed that this problem can be partly mitigated by matching acquired data with data obtained from Monte Carlo simulations that have been performed with varying model parameters and stored in a database. Other studies have also encountered the concept of barrier-limited exchange, but discussed it in other terms [78, 95, 96]. The membrane permeability at which the exchange is no more barrier limited also represents the point at which increased permeability results in increased ADC values, as shown in Fig. 6 [97].

Under conditions in which the exchange is not barrier-limited, but rather limited by the time necessary to diffuse across the cell, the exchange time in a cylinder is given by [93]

13 $$ \tau_{\rm i} = d^{2} /32D_{\text{intra}} + \, d/4P_{\text{d}} , $$

rather than by Eq. 12, with (*V*/*A*)_i_ = *d*/4 for a cylinder.

### Summary of models

Table 1 shows a summary of the models describing RS in white matter, although these models could equally well be employed to describe *S* independently of the diffusion encoding direction in isotropic systems. Expanding the models is generally straightforward as for example the inclusion an isotropic CSF component [67]. The models could also be combined, for example, to model exchange, a diameter distribution, and orientation dispersion, using seven model parameters to describe RS. Accurate representation of the white matter microstructure probably requires all these features to be present in the model. In addition, two or three hindered and restricted components with different orientations are required to model the diffusion in white matter regions that contain multiple fibre populations with different orientations. Behrens et al. [98] suggested that at least a third of all white matter voxels contain more than one fibre population. Potentially, nearly all white matter voxels may contain crossing fibres [99].

**Table 1:** Summary of models describing RS in white matter, where *n* denotes the number of model parameters

Model name	Model parameters	*n*	Comments
Very minimal	*S*_0_, *f*_r_, RD_h_	3	Assumes RD_r_ = 0
Compartment	Minimal + *d*	4	Similar to the CHARMED model [20]
Diameter distribution	Minimal + *d*, *σ*_d_	5	Similar to the AxCaliber model [21]
Two-compartment exchange	Compartment + *τ*_i_	5	Based on the Kärger equations
Orientation dispersion	Compartment + *κ*	5	Model by Zhang et al., also requires AD, which can be assumed to be equal to *D*_i_
Undulation	Minimal + *A*, *L*, AD	6	RS given by a propagator model [80]
FEXI	ADC, *σ*, AXR	3	In addition to these three model parameters, *S*_0_ is included for each mixing time
DKI	*S*_0_, RD, RK	3	The full diffusional kurtosis model uses 22 model parameters [26]
Biexponential	*S*_0_, *f*_s_, *D*_f_, *D*_s_	4	Modelling a fast and a slow diffusion tensor requires 14 model parameters

All models could be extended to describe the signal in any direction by using three more model parameters that define the direction of the fibre (*θ*, *φ*) and the axial diffusivity (AD). The FEXI, diffusional kurtosis, and biexponential models are included for comparison

Model selection is not a trivial matter, because clearly the microstructure of the white matter is highly complex in most if not all parts of the brain. Estimating all properties of all fibre populations may not even be possible, so simplifications are required. The NODDI model by Zhang et al. [82] is a good example of where simplifications allows more precise estimates of relevant parameters, but model simplification requires approximations that may be invalid. For example, the assumption of non-exchanging compartments is invalid in sub-acute ischemic stroke lesions [100]. However, the use of more complex models, having a greater number of model parameters, is not always feasible, since fitting the model parameters may capture features of the signal noise rather than underlying microstructure. To avoid overfitting, testing whether the data support a complex model over a simple model can be done with an *F* test, for example, as performed by Kiselev and Il’yasov [101]. They showed that the kurtosis model (three parameters) could be used just as well as the biexponential model (four parameters) to fit data acquired in vivo with high *b*-values in 20–41 % of the grey matter voxels investigated. This means that not all of the data acquired supported the biexponential model. The Bayesian information criterion can also be used to compare models. Using data acquired in the corpus callosum of perfusion-fixated rat brains, Panagiotaki et al. [102] evaluated 47 analytic models of diffusion in multiple non-exchanging compartments with up to 11 model parameters. They found that models incorporating an intra-axonal component having restricted diffusion generally explained the data better than models assuming hindered diffusion in all components. However, diffusion MRI data alone may be insufficient to select between models of equal complexity. For models having an equal number of model parameters, these may be transformed from one model to the other. For example, the number of model parameters in the very minimal model (three) is equal to that of the kurtosis model (for measurements performed in a single direction). Consequently, the parameters in the two models can be related according to *f*_r_ = RK/(RK + 3) and RD = *f*_h_·RD_h_, where RK is the radial kurtosis [30]. Three of the models in Table 1, the diameter distribution model, the two-compartment exchange model, and the orientation dispersion model, all describe the signal curves using five model parameters. Finding the optimal model in such a case requires careful model evaluation [102]. Choosing the optimal model could also be aided by the contribution of independent external information, for example, that acquired by microscopy. The FEXI protocol could also contribute with independent information regarding exchange, since it is sensitive specifically to the exchange between the slow and fast diffusion components [87, 103].

### Extracellular diffusion

In addition to the concepts included in the models above, the structure of the extracellular space will also influence the water diffusion. The extracellular space is tortuous, which in nerves results in diffusion that is more hindered in the direction perpendicular to the nerve than parallel to it, according to

14 $$ {\text{RD}}_{\text{h}} = {\text{AD}}_{\text{h}} /\lambda^{2} , $$

where *λ* is the tortuosity factor. For ion diffusion in the rat cerebellum, this factor has been measured as *λ* = 1.55 ± 0.05 [104], however, the value of *λ* depends on the fractional volume of the extracellular space (*v*_extra_). For example, Lipinski et al. [105] reported that $\lambda = v_{\text{extra}}^{ - 0. 4 1}$, based on particle simulations on digitised images of histological sections. Other relations have also been employed, for example *λ* ^2^ = 1 + (1 − *v*_extra_)^3/2^ by Hall et al. [106] and $\lambda^{ 2} = v_{\text{extra}}^{ - 1}$ by Alexander [67].

By using a model that relates *λ* and *v*_extra_, the number of model parameters may in some cases be reduced by one, since Eq. 14 relates RD_h_ to AD_h_. However, the relation between *λ* and *v*_extra_ is uncertain and is likely to be influenced also by factors other than *v*_extra_, such as the narrow spaces between cells [107]. In addition, the hindered fraction *f*_h_ may be an inaccurate proxy of *v*_extra_, since it may represent water from both the extracellular space and from cells in fast exchange with it [49]. Equation 14 may thus be more suitable for post-hoc analysis of estimated model parameters than for incorporation in biophysical models.

### Model fitting

The diffusion MRI experiment is relatively simple to describe from a theoretical point of view, but implementing it and analysing the results is more complicated in practice, as described thoroughly elsewhere [108, 109]. The most important aspect to consider in the context of biophysical modelling of white matter diffusion is the statistical distribution of the MRI signal. For single-receiver systems, the magnitude signal is Rice-distributed [110, 111]. This distribution is approximately Gaussian if the SNR, defined by SNR = S/*σ* with σ being the standard deviation of the signal in the real or imaginary channel, is higher than approximately two, but has an expectation value of *σ*(*π*/2)^1/2^ when the true signal is zero. This signal bias is known as the rectified noise floor. If *σ* is known, the Rice distribution can be taken into account in the model fitting as shown, for example, by Veraart et al. [112] for the kurtosis model. Multiple receive coils and parallel imaging, techniques widely used today, results in an approximately non-central chi distributed rather than a Rice distributed signal [113–115]. The noise level is also non-uniform across the image volume when multiple receive coils are used [116]. Post-processing such as motion correction also affect the signal distribution [115]. The noise floor bias, which is present also when multiple coils and parallel imaging is used [113, 114], can make it challenging to distinguishing a water signal from environments with highly restricted diffusion (*D*_r_ ≈ 0) from the level of the noise floor. Knowledge of the level of the noise floor is thus important in the model fitting.

## Model validation in cell suspensions and excised tissue

Model development has been accompanied by validation experiments in suspensions of, for example, red blood cells and yeast cells. Before comparing in vivo and in vitro results, however, differences in water temperatures could be important to consider since *D*_bulk_ and presumably also *D*_intra_ increase by approximately 50 % when the temperature increases from 20 to 37 °C [117]. Measurements at low temperatures are thus beneficial in terms of the resolution limit: in order to keep *D*_r_, *α*, and *β* equal at the two temperatures, the values of *δ* and *t*_d_ at 37 °C should be two thirds of those at 20 °C (Eq. 5). In order to preserve *b*_max_, the value of *g*_max_ would then need to be approximately 80 % greater at the higher temperature.

Diffusion experiments on excised tissue provide an opportunity to compare model-based estimates of structural parameters of the tissue with independent histology-based estimates. For conclusions drawn from results obtained in excised tissue, the time interval between death and tissue fixation should be considered since it influences diffusion in neural tissue. For instance, the MD in the corpus callosum in a dead brain is reduced from approximately 0.17 to 0.06 μm^2^/ms during two weeks of brain decomposition [118]. Studies of human tissue are particularly sensitive to this issue, in contrast to animal tissues that may be fixed directly postmortem, or premortem by perfusion fixation. Fixation itself also affects the diffusion; for example, it reduces MD but not FA [119, 120]. Moreover, differences in diffusivity between infarcted and healthy tissue are lost during fixation [119]. The storage time of the fixed tissue only has a minor influence on the MD and FA [121].

### Studying exchange using red blood cells and yeast cells suspensions

The exchange rate in red blood cells has been determined using various independent methods such as diffusion NMR and the Kärger model [117, 122, 123], the Mn^2+^ doping ^1^H NMR method [124], and studies of diffusion using internal magnetic field inhomogeneity [125]. The different methods have provided similar results. The diffusional membrane permeability of the mammalian red blood cell is high, with *P*_d_ in the range 49–112 μm/s at 37 °C, as measured in various species [124]. The high values of *P*_d_ in combination with the small sizes of red blood cells lead to values of *τ*_i_ in the order of 5–10 ms according to Eq. 12, assuming *V*/*A* ≈ 0.5 μm [91].

The two-compartment exchange model has been used to quantify *τ*_i_ in erythrocyte ghost models. As expected, blocking of the aquaporin channels results in increased values of *τ*_i_ [123]. It has also been shown that the value of *f*_r_ estimated from diffusion data is lower than that obtained with an independent method [117]. This underestimation might be expected, since the exchange is not barrier-limited for the high membrane permeability found in red blood cells.

Yeast cells provide a relatively simple model system for diffusion NMR and MRI investigations, in which the exchange rate is much slower than in red blood cells. Åslund et al. [13] used the double PGSE sequence to map the exchange rate in yeast cells, and showed that *P*_d_ is dependent on the temperature. Suspensions of yeast cells were used to validate the FEXI model and to compare results obtained with NMR spectrometers and those obtained using a clinical MRI scanner [87]. The results from both platforms resembled each other and agreed with expectations from other studies.

### Intracellular diffusion

Independent estimates of *D*_intra_ are valuable in the construction and application of biophysical models of diffusion in tissue. Zhao et al. [126] performed measurements with very short diffusion times and reported *D*_intra_ = 2.0 ± 0.3 μm^2^/ms in HeLa cells with diameters of approximately 20 μm, compared to *D*_bulk_ ~ 3 μm^2^/ms for free water at 37 °C. In another study, Beaulieu and Allen measured the intra-axonal diffusion coefficient in giant axons of the squid, which are large enough (200–1,000 μm) to allow for measurements of intra-axonal diffusion coefficients unaffected by restriction effects of the membranes (i.e. *α* → 0 and *β* → 0 in Eq. 7). The values measured were AD_intra_ = 1.61 ± 0.06 μm^2^/ms and RD_intra_ = 1.33 ± 0.09 μm^2^/ms, respectively, which can be compared to *D*_buk_ = 2.08 ± 0.04 μm^2^/ms for free water at 20 °C [56]. Longitudinally ordered neurofilaments within the axons were suggested as the cause of the small anisotropy, i.e., the difference in axial and radial diffusivity of the intra-axonal water. Anisotropy of the intra-axonal diffusivity would likely have some impact on axon diameter estimated obtained by analyses performed with the two-compartment model on data obtained with clinical MRI scanners, since the value of RD_intra_ influence *α* and *β*. However, the impact would likely be limited. In both of these studies, *D*_intra_ ~ 2/3 *D*_bulk_, but it is even lower in yeast cells [127].

The diffusivity within cells might be inhomogeneous. Sehy et al. [128] showed ADC values in the *Xenopus* oocyte ranging from 0.5 μm^2^/ms in the vegetal pole to 1.7 μm^2^/ms in the nucleus. In neural tissue where the cells are up to three orders of magnitude smaller than the millimetre sized oocyte, such an inhomogeneity probably contributes less to the value of *D*_r_ than the size of the cell. Galons et al. [129] investigated rat glioma cells and reported that 50–60 % of the intracellular water has slow diffusion, which also showed evidence of being restricted. This could potentially confound results of model-based analysis that assume a homogeneous intracellular environment, and requires further investigation.

### Excised nerves

**Fig. 7:**
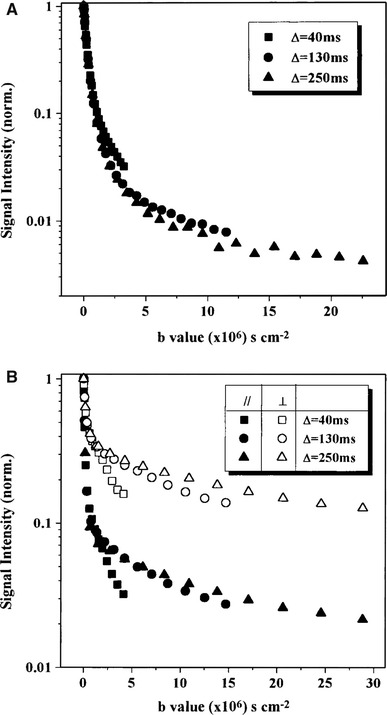
Normalized attenuation of water signal as a function of the diffusion time, averaged over three brains (**a**), and three nerves (**b**). *Full* and *open symbols* represent nerve data in which the diffusion gradient direction was parallel (AS) and perpendicular (RS) to the long axis of the nerve, respectively. In the brains (*top*), the slope of the slow component increase with prolonged diffusion times, while the slope of the slow component is reduced for prolonged diffusion times in nerves (*bottom*). These two phenomena are the hallmarks of exchange and restricted diffusion, respectively. Note the unit of *b*, where 10^6^ s/cm^2^ = 10^4^ s/mm^2^ = 10 ms/μm^2^. Reproduced from Assaf and Cohen [133] with permission from John Wiley and Sons

Investigations of diffusion in excised tissue using the single PGSE sequence have been performed in several studies, the first of them in 1970s [130, 131]. Most studies have investigated optic and sciatic nerves, spinal cord, and whole brain. Signal-versus-*b* curves acquired in excised nerves are multi-exponential for diffusion encoding performed both perpendicular and parallel to the nerves [22, 132, 133]. The fast diffusion component has been reported to be almost independent of the diffusion time, while the slow diffusion component has shown evidence of being restricted (Fig. 7). The fast and slow diffusion components were accordingly assigned to the extracellular and intra-axonal spaces, respectively [133]. Estimates of the axon diameter distribution using the AxCaliber model have shown good agreement with corresponding histology-based estimates in porcine optic and sciatic nerves [21]. The estimates were based on several sets of diffusion measurements acquired perpendicular to the nerve and with diffusion times between 10 and 80 ms.

Parameters correlating with the axon diameter can also be obtained using model-free approaches, for example, *q*-space analysis [66, 134, 135]. However, *q*-space analysis underestimates compartment sizes unless *δ* < 0.02 *d* ^2^/*D*_intra_ [64], which corresponds to *δ* < 80 μs for *d* = 2 μm. Experiments in excised nerves have verified that the compartment size estimated from the slow diffusion component depends on *δ* [136], as expected from Eqs. 5 and 6.

**Fig. 8:**
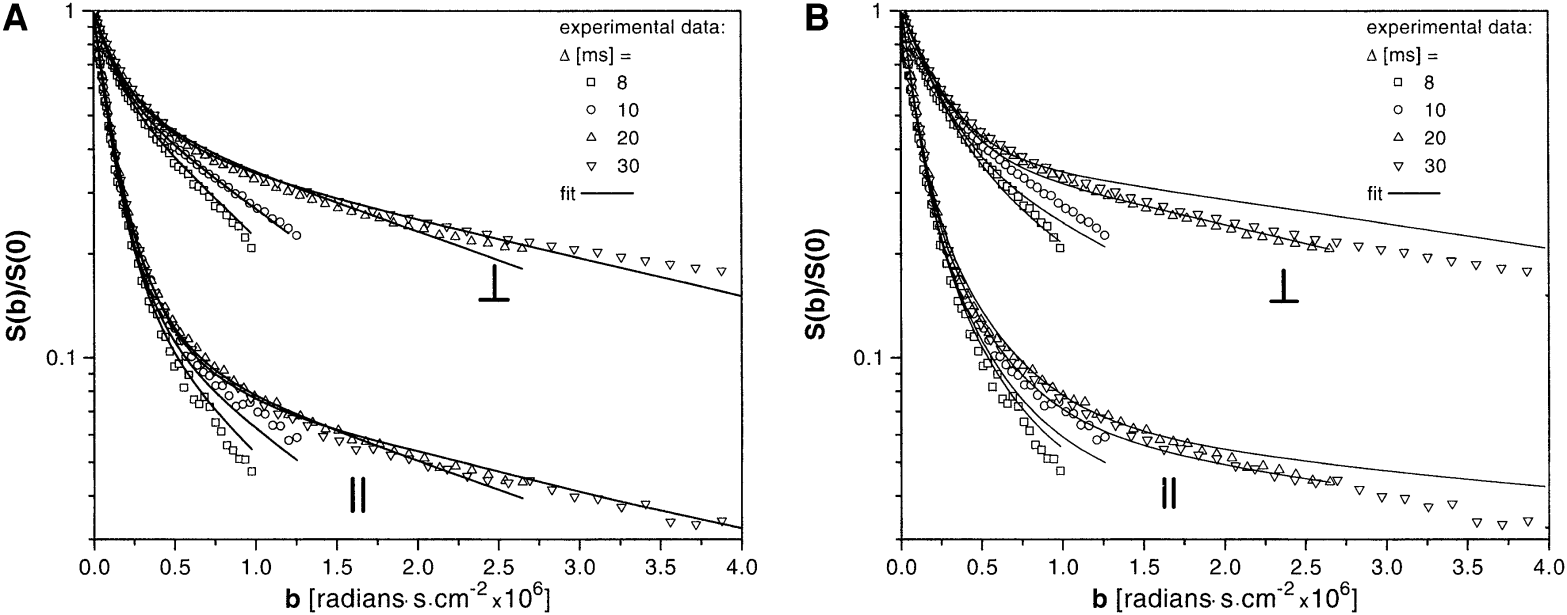
Signal-versus-*b* curves obtained with diffusion encoding perpendicular and axial to the bovine optic nerve. **a** The global fit of a three-pool tissue model (*solid lines*) to the experimental data (data points). **b** The results of the three-pool model without permeability (*P* = 0 for all pools). The misfit for high *b* values is observable. Note the unit of *b*, where 10^6^ s/cm^2^ = 10^4^ s/mm^2^ = 10 ms/μm^2^. Reproduced from Stanisz et al. [22], with permission from John Wiley and Sons

Water exchange between the intra-axonal and the extracellular space has been investigated by, for example, Stanisz et al. [22] who modelled nervous tissue as consisting of permeable and uniformly-sized spheres and parallel ellipsoids. The spheres represented glia cells and the ellipsoids represented axons, assuming that the diffusion was restricted also in the direction parallel to the axons. Based on measurement in the bovine optic nerve, the authors found that the model required a non-zero membrane permeability (Fig. 8), which was estimated to be *P*_d_ = 9 ± 2 and 17 ± 3 μm/s for the axon and glial membrane, respectively. This corresponded to exchange times of approximately 30–60 ms. The axonal and glial water volume fractions were 17 ± 4 and 43 ± 5 %, respectively.

Results from other studies also indicate that effects of water exchange are detectable in diffusion-weighted data acquired in excised nerves. Bar-Shir and Cohen performed bi-gaussian analysis of the propagator, similar to biexponential analysis of the signal-versus-*b* curve, and demonstrated that *f*_s_ is reduced as *t*_d_ is prolonged above 10 ms in measurements on the swine optic and sciatic nerves [136]. The observation was attributed partly to water exchange. Biton et al. [137] observed similar trends in normal spinal cord. The authors also investigated myelin-deficient spinal cord, where the root-mean-square displacement of the slow diffusion component increased almost linearly with (*t*_d_)^1/2^, for *t*_d_ between 22 and 200 ms. This observation suggests higher exchange rates in the myelin-deficient spinal cord than in the normal one, as could be expected. Assaf et al. [133] observed reduced values of RS at high *b*-values for prolonged diffusion times in the spinal cord of the 7-day-old rat, which is evidence of exchange (Fig. 5). In the mature spinal cord, however, the values of *RS* increased for prolonged *t*_d_, as expected for restricted diffusion. In summary, exchange in excised nerves appears to be fast enough to affect the signal curves acquired so that exchange should be included in models of white matter diffusion. Measuring the exchange rate may be just as important as measuring the axon diameter, since it is altered both in disease and during development.

## Model validation in vivo

**Fig. 9:**
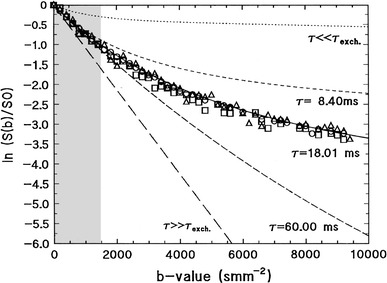
The plot shows signal attenuation curves obtained in vivo, in the striatum and the cortex of the rat, for three different diffusion times (8.4, 18.01, and 60 ms shown by *squares*, *circles*, and *triangles*, respectively). The *curves* obtained show no diffusion time dependence, in contrast to the dashed curves that would have been expected from the a two-component model (similar to the two-compartment model, but with a fixed diffusion coefficient of the slow component). Note the unit of *b*, where 1 s/mm^2^ = 10^−3^ ms/μm^2^. Reproduced from Niendorf et al. [53], with permission from John Wiley and Sons

**Fig. 10:**
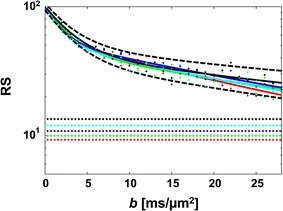
RS-versus-*b* curves acquired in the corticospinal tract of a healthy volunteer. Five curves were acquired with *t*_d_ from 64 to 256 ms. *Solid lines* are biexponential fits. No obvious effects of a varied diffusion time is observed in the signal curves. *Dashed lines* indicate ± 1 standard deviation of the signal acquired with *t*_d_ = 256 ms. *Dotted lines* show the mean noise level. Reproduced from Nilsson et al. [23], with permission from Elsevier

In contrast to the case in excised tissue, the signal-versus-*b* curves observed in vivo are conspicuously independent of *t*_d_, as reported for measurements performed in regions such as the cortex and striatum of the rat as shown in Fig. 9 [53], human white and grey matter, [52] and white matter of the cat [138]. Investigations of RS in the corticospinal tract for diffusion times between 64 and 256 ms with *b*_max_ = 28 ms/μm^2^ showed no effects of a varied diffusion time (Fig. 10) [23]. At a first glance, these results seem to contradict the assumption that the slow diffusion fraction is restricted, especially since the reported values of *f*_s_ are generally much lower than the value of 80 % that would be expected if all intracellular water molecules were restricted in their diffusion. To resolve these issues, it is helpful to analyse white and grey matter separately and to investigate four concepts one by one: differences in relaxivity between excised and living tissue, expected values of the signal fractions, effects of restricted diffusion, and the rate of compartmental exchange.

### Relaxivity and diffusion

Studies of the transversal relaxation in excised nerves have provided evidence of three water components, assigned to myelin water, extracellular water, and intra-axonal water, with *T*2 relaxation times of 10–20, 65–80, and 250–350 ms, respectively, where the specific values depend on the magnetic field strength [77, 139]. Other studies have suggested that the *T*2 relaxation time is longer in the extracellular space than in the intra-axonal space [139, 140]. However, this assignment is not supported by diffusion experiments showing that *f*_r_ increases for prolonged TE [77, 133]. Most in vivo studies of transversal relaxation rates have observed two components with short (10–50 ms) and long (70–130 ms) *T*2 relaxation times, assigned to myelin water and the combined contribution of intra- and extracellular water [141–143]. Support for three components *in vivo* have been found in the peripheral of the amphibian *Xenopus laevis* [144], and in some regions in the human brain [141]. Three components could be interpreted as significantly longer *T*2 relaxation times for extracellular compared to intra-axonal water [142]. However, results from diffusion MRI studies in the CNS suggest at most a negligible difference in transversal relaxation between the intra-axonal and extracellular space in vivo: the fast- and slow diffusion components have indistinguishable relaxivities [145, 146], DTI metrics are insensitive to TE [147], and biexponential model parameters are insensitive to TE within practically achievable ranges [52]. These observations suggest that the signal fractions in vivo do reflect the relative volume fractions of the various diffusion components, independent of echo and repetitions times within feasible ranges. Fast exchange between intra-axonal and extracellular water would also render their relaxivities inseparable; however, such a fast exchange is unlikely in healthy white matter.

### Signal fractions

Several authors have performed high *b*-value diffusion experiments in vivo and quantified the resulting signal-versus-*b* curves using a biexponential model. Most of these studies have yielded values for *f*_s_ in the range 20–35 % [52, 53, 138, 148–150]. This range covers results from varying protocols, acquired in rats as well as in humans and in grey- as well as white matter or a combination of both (Table 2). Values outside this range have been found in studies of white matter where the signal-versus-*b* curve was acquired in a well-controlled direction compared to the direction of the axons. For instance, Clark and Le Bihan reported *f*_s_ ≈ 50 % in the internal capsule, for diffusion encoding performed in the left-right direction [52]. Nilsson et al. [23] similarly reported *f*_s_ ≈ 50 % for measurements performed perpendicular to the corticospinal tract.

**Table 2:** A summary of the fast and slow ADCs (*D*_f_, *D*_s_) obtained using the biexponential model in rat and human brains, together with the slow diffusion fraction and details of the protocols employed (*t*_d_/δ, *b*_max_)

Tissue	Reference	D_f_/D_s_ (μm^2^/ms)	*f*_s_ (%)	*t*_d_/*δ* (ms)	*b*_max_ (ms/μm^2^)
Rat brain, WM + GM	Niendorf et al. [53]	0.84/0.17	20	18/n/a	10
Rat brain, WM	Ronen et al. [138]	0.69/0.08	~30	10/8.5	12.5
Rat brain, WM + GM^a^	Pfeuffer et al. [146]	0.70/0.08	n/a	60/7	20
Human brain, WM^b^	Clark et al. [150]^c^	0.75/0.30	37	25/27	3.5
Human brain, WM	Clark et al. [52]	1.12/0.16	34	25/n/a	4
Human brain, WM^d^	Maier et al. [149]^e^	1.25/0.16	36	35/35	5
Human brain, WM + GM	Mulkern et al. [148]	1.40/0.25	26	56/80	6
Human brain, WM	Nilsson et al. [23]	0.45/0.03	51	64/50	28
Human brain, thalamus	Clark et al. [150]^c^	0.76/0.45	37	25/27	3.5
Human brain, thalamus	Maier et al. [149]^e^	1.18/0.23	32	35/35	5
Mouse cortex	Schwarz et al. [189]	0.77/0.18	21	13/8	10
Mouse cortex, ischaemic	Schwarz et al. [189]	0.58/0.13	43	13/8	10
Mouse cortex, cold-injured	Schwarz et al. [189]	0.89/0.10	33	13/8	10
Adult rat, post mortem	Niendorf et al. [53]	0.51/0.09	31	18/n/a	10

Entries are ordered by category and *b*_max_

^a^Based on two linear regressions

^a^Averaged over corpus callosum, the internal capsule, frontal white matter and centrum semiovale

^c^Dual tensor model, based on two linear regressions

^d^Averaged over the corpus callosum and the internal capsule

^e^Dual tensor model

The hypothesis that the slow diffusion component represents intracellular water has been challenged by the fact that the total intracellular volume fraction (*v*_intra_) is much higher than the values reported for *f*_s_ [52, 149]. However, intracellular water is distributed in several different environments such as cell bodies of neurons and glial cells as well as in axons and dendrites. Diffusion measured in parallel with axons or dendrites will have a high diffusivity and appear to be unrestricted. Moreover, astrocytic water is probably in fast exchange with the extracellular water, since the ADC is reduced by up to 50 % when the astrocytic AQP4 expression is reduced [49]. Parts of the intracellular water fraction may thus show fast diffusion.

Some intracellular water, such as myelin water, is MR-invisible at the echo times by which most diffusion experiments are performed with clinical MRI scanners. The fractional myelin volume (*v*_myelin_) may nevertheless have an influence on *f*_r_ due to geometrical reasons [55]. Assuming that water compartments other than the intra-axonal, extracellular and myelin compartments are negligible, we have *v*_axon_ + *v*_extra_ = 1 − *v*_myelin_, where *v*_extra_ here is the fractional volume of the extracellular space and other spaces in fast exchange with it. The relation between *v*_axon_, *v*_extra_ and *v*_myelin_ can be simplified by assuming that the ratio between the axonal outer and inner diameters (*g*) is independent of the axon diameter (Fig. 1b), and that axons are cylindrical, so that *v*_axon_ = *g* ^2^ (*v*_axon_ + *v*_myelin_) = *g* ^2^ (1 − *v*_extra_). Assuming *v*_extra_ = 20 % and *g* = 0.65 [40, 151], the expression for *v*_axon_ evaluates to 60 %. In the spinal cord, results from segmented histology images suggest that *v*_axon_ may be as low as 45 % [135, 152]. Assuming that the water concentrations and relaxivities in the intra-axonal and extracellular spaces are approximately equal, the expected value of *f*_s_ may thus be in the range 45–60 %. The presence of axonal orientation dispersion may further reduce the value of *f*_r_ [80]. Since *f*_s_ ≈ 50 % for diffusion measured perpendicular to white matter [23], it might thus plausible to associate the slow diffusion component to intra-axonal water in white matter, also for the in vivo case. Corresponding analysis of grey matter is more complicated, due to the large dendritic orientation dispersion [153].

### Restricted diffusion

**Fig. 11:**
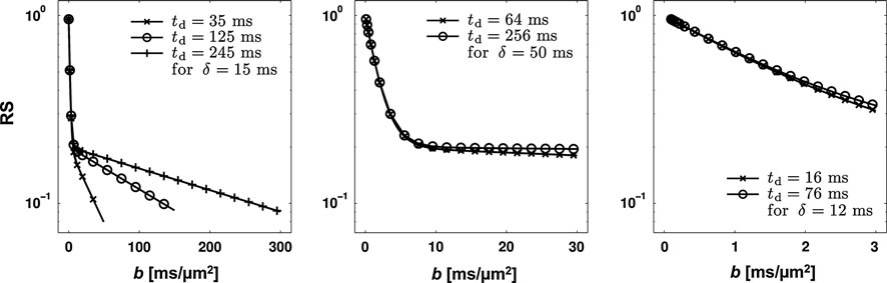
RS-versus-*b* curves produced using the compartment model with *f*_r_ = 0.2, *d* = 6 μm, RD_h_ = 0.6 μm^2^/ms. The protocols in the *left*, *middle* and *right panel* intend to resemble the protocols employed by Assaf et al., by Nilsson et al., and by Alexander et al., respectively. Note the differing scales on the *x*-axis

In contrast to what is the case in excised nerves and also expected for restricted diffusion in white matter, the *RS*-versus-*b* curves obtained in vivo are generally independent of *t*_d_ [23, 52, 53, 138]. DTI metrics, obtained in the corpus callosum, are also independent of *t*_d_ between 8 and 80 ms [154]. However, specialised diffusion MRI measurements by Does et al. [155] have revealed a *t*_d_ dependence of the ADC for diffusion times below approximately 5 ms. Taken together, these results may imply that RD_r_ ≈ 0 for diffusion times longer than approximately 5 ms. In such cases, the absence of a diffusion-time dependence in RS is to be expected. This is exemplified in Fig. 11, where the compartment model was used to generate RS(*b*), assuming *d* = 6 μm and protocols that resemble those employed in NMR spectrometer-based investigations of excised tissue with those used at clinical MRI scanners [23, 67, 133]. While the *t*_d_ dependence of the signal is evident for the spectrometer case, it is much weaker for the two cases corresponding to clinical scanners. Specifically, RD_r_ ≈ 0 at both diffusion times in the protocol of Nilsson et al. [23], due to the high value of *δ* featured in that protocol. The value of *δ* is much shorter in the protocol resembling that employed by Alexander et al., but the low value of *b*_max_ results in only a small signal difference between the two diffusion times.

In apparent contradiction with the assumption that RD_r_ ≈ 0, biexponential quantification of signal-versus-*b* curves shows that *D*_s_ is significantly higher than zero in vivo [52, 53, 138, 148–150]. Values of *D*_s_ above zero could, however, be expected for measurements performed with the diffusion encoding not being exactly perpendicular to the nerve. If RD_r_ ≈ 0 and the encoding direction deviates by an angle *ϕ* from the plane with normal **u** (the direction of the nerve), we would expect

15 $$ D_{s} = \sin^{2} (\phi){\text{AD}}_{\text{r}} . $$

This means that the value of *D*_s_ observed by Nilsson et al. [23], using a 3-T head scanner, could have been obtained if *ϕ* ≈ 10°, i.e., if the estimated direction of the nerve deviated more than ten degrees from its true value. Fibre orientation uncertainty can be estimated [156], but are not available for the study. We may however note that such a large deviation appears to be unlikely in a region with high FA [156], which was 0.72 ± 0.03 in the region assessed by Nilsson et al. [23]. The high values of *D*_s_ observed in vivo probably demands other explanations.

Two other hypotheses could explain the non-zero value of *D*_s_ and the apparent absence of a *t*_d_-dependence of RS(*b*) at high *b*-values. Nilsson et al. [23] suggested that this could be the effect of exchange between the intra-axonal and extracellular space. This hypothesis will be discussed in the next section. Nilsson et al. [80] also described effects of axonal undulation on RS(*b*), assuming RD_r_ = 0, and reported that macroscopic undulation results in *t*_d_-insensitive and apparently biexponential signal-versus-*b* curves with non-zero values of *D*_s_.

Despite the uncertainties regarding the biophysical mechanism responsible for the slow diffusion component in vivo, estimates of the axon diameter from diffusion MRI data acquired in vivo correlate with corresponding estimates from histology images. Using the AxCaliber model, which assumes impermeable, straight and parallel axons, Barazany et al. [157] estimated the axon diameter distribution from data obtained from the corpus callosum in the rat brain, using a system with *g*_max_ = 400 mT/m. The known variations in the axon diameter distribution along the corpus callosum from the anterior (genu) to the posterior (splenium) were largely reproduced, although the axon diameter distributions found by AxCaliber were generally broader than those obtained by histology. The authors suggested that this deviation was caused by tissue shrinkage during histological preparation. Moreover, the reported values of *f*_r_ were in the range 15–30 %, which is lower than expected. Alexander et al. [67] similarly showed an agreement between the known variations in axon diameter along the corpus callosum and an axon diameter index estimated from diffusion MRI data acquired in two fixed monkey brains and two live volunteers, using an animal experimental system (*g*_max_ = 140 mT/m) and a clinical MRI scanner (*g*_max_ = 60 mT/m), respectively. The term “axon diameter index” refers to a summary statistic over the axon diameter distribution that may differ from the volume-weighted average axon diameter, possibly due to non-linear weighting effects when the compartment model assuming a single diameter is used (Fig. 3). The index was, however, overestimated both in the monkey case and the human case, as compared to the value expected from histological investigations.

### Exchange

Several authors have investigated the intracellular exchange time in live brain tissue and reported values between approximately *τ*_i_ = 25 and 620 ms [93, 146, 158, 159]. The results were obtained from large volumes containing contributions from a mixture of grey and white matter. Nilsson et al. [23] reported an intra-axonal exchange time of *τ*_i_ = 306 ± 45 ms in a well-defined region of the corticospinal tract. Although this value of *τ*_i_ is within the range suggested by previous studies, the analysis did not account for the likely presence of orientation dispersion [23]. The presence of orientation dispersion would probably result in an underestimated value of *τ*_i_ when analysing the data using the two-compartment exchange model, since effects of exchange and of orientation dispersion on RS are similar. The water exchange rate in the human brain has also been investigated using filtered exchange imaging (FEXI), which yields the so-called apparent exchange rate (AXR). In regions of interest placed in frontal and parietal white matter, as well as in the internal capsule, the AXR was 1.6 ± 0.11, 1.0 ± 0.12, and 0.8 ± 0.08 s^−1^, respectively [103]. These AXR values correspond to exchange times of between 1.25 and 2.5 s, assuming *f*_r_ = 50 %. These estimated values of τ_i_ are considerably longer than those suggested in previous studies.

On the lower part of the exchange-time range observed in the brain, values of τ_i_ between 25 and 135 ms were obtained in grey and white matter regions by Pfeuffer et al. [92, 146] based on constant gradient experiments and reported in two separate studies. These results could suggest the presence of a fast exchanging component with exchange times in the order of 10–100 ms. While the intra-axonal water is presumably in slow exchange with the extracellular space (a slow diffusion component is observed also at long diffusion times), the exchange rate in astrocytes could be high. A reduction of the membrane permeability of these cells, using RNA interference to knockout aquaporin expression, results in ADC reductions of approximately 50 % [49]. Such an effect is only to be expected if the initial exchange rate is high (Fig. 6). Does et al. [160] similarly suggested that one of the components in the *T*2 spectrum originated from water outside myelinated axons, but within compartments in rapid exchange with the extracellular space.

The rate of water exchange between the intra-axonal and extracellular spaces is probably strongly influenced by the myelin sheath (Fig. 1). For example, studies analysing the relaxivity of different components have suggested that exchange between myelin water and water in the intra-axonal and extracellular space occurs with exchange times of approximately 100–200 ms [143, 161]. Some studies have assumed that the overall permeability of myelin is inversely proportional to the thickness of the myelin sheath [162, 163]. However, this assumption may only be valid for thin membranes [164]. The intricate structure of the sheath suggests that there could be multiple mechanisms by which the properties of myelin influence the exchange rate. For example, the periaxonal space is connected to the extracellular space, so that water molecules crossing the axolemma can reach the extracellular space without having to pass the myelin membranes (Fig. 1). Another mechanism has been investigated using simulations, in which the myelin was assumed to be impermeable, but where exchange was allowed to take place at the nodes of Ranvier (Fig. 1), as presented in a conference abstract [165]. Describing the nodes by their width (*w*) and internode distance (*L*), the ratio of permeable surface to the total volume is given by [103]

16 $$ A/V = 4w/{\text{d}}L, $$

which gives an intra-axonal exchange time of *τ*_i_ = d*L*/4*wP*_d_. Although this model is inaccurate for large values of *L*, it may be used to deduce that larger axons with larger distances between the nodes of Ranvier would be expected to show lower exchange rates than thin axons with short distances between the nodes. Future studies could investigate this model by determining the exchange rate in maturing white matter. Simulations suggest that this mechanism would render intra-axonal exchange times in the order of seconds or longer [165], which lends credibility to the idea that intra-axonal water is in slow exchange with the extracellular space.

## Application: Ischemic stroke

Several authors have investigated how DTI parameters are influenced by ischaemic stroke at various stages after onset and hypothesised about the cause of these alterations, as reviewed by Sotak [166]. High *b*-value investigations of diffusion in stroke lesions are less abundant than corresponding DTI studies, but a few studies have quantified the signal-versus-*b* curve using the biexponential model. Schwarcz et al. [167] showed that *f*_f_ decreases in the hyperacute stage of global ischaemia in the mouse brain, as could be expected from the cell-swelling hypothesis that predicts a reduction of the amount of extracellular water in stroke lesions. In addition, both *D*_s_ and *D*_f_ were reported to decrease as compared to the normal case. Brugières et al. similarly found that *f*_f_ decreased in subacute stroke lesions in a patient group, but found that *D*_s_ and *D*_f_ increased and remained unaffected, respectively [168]. These conflicting results could possibly be explained by the different time from onset in the two studies.

**Fig. 12:**
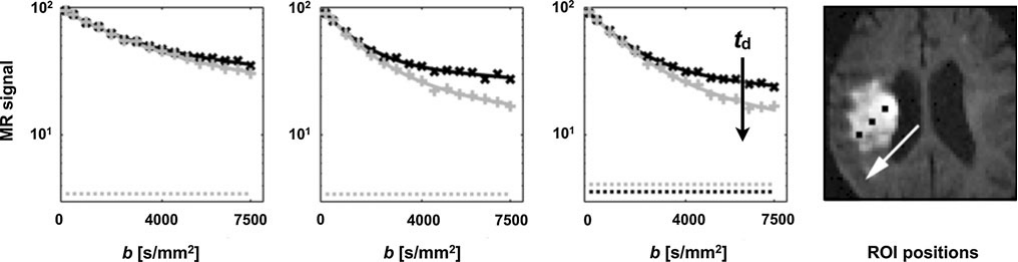
Signal-versus-*b* curves obtained with *t*_d_ = 60 ms (*black*) and 260 ms (*grey*), from three regions of interest shown on top of a DWI image on the right, where the *white arrow* indicates the order of the panels from *left* to *right*. Measurements were performed approximately 30 h after onset. *Dashed lines* represent the noise floor. Clear evidence of exchange is seen in the *middle* and *right* panel, as reduced signal values for prolonged *t*_d_. Note the unit of *b*, where 1 s/mm^2^ = 10^−3^ ms/μm^2^. Reproduced from Lätt et al. [100], with permission from John Wiley and Sons

**Fig. 13:**
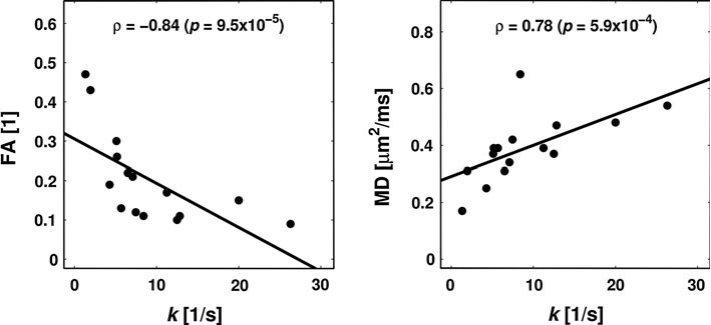
Correlation plots for data obtained from Lätt et al. [100], showing the correlation between *k* = 1/*τ*, and FA and MD in the *left* and *right panels*, respectively. Correlation was significant for both plots, based on a Spearman correlation test. The *solid line* is the linear fit

The presence of water exchange between the fast and slow diffusion components complicates the interpretation of results from biexponential analysis of signal-versus-*b* curves. Sub-acute stroke lesions were investigated using high *b*-values and two different diffusion times by Lätt et al. [100]. Effects of exchange were clearly visible in most lesions (Fig. 12). The two-compartment exchange model was fitted to signal curves geometrically averaged across the diffusion-encoding directions, thereby implicitly assuming that the underlying tissue was isotropic. While this assumption is invalid for healthy white matter, the sub-acute stroke lesions investigated showed lower FA values than healthy tissue. The reduction in anisotropy is also present at high *b*-values, as observed by a reduced kurtosis anisotropy in hyper-acute and acute stroke lesions [169]. By performing an extended analysis of the values presented by Lätt et al. [100], a significant correlation is found between *k* = 1/*τ*_i_ and both FA and MD (Fig. 13). In addition, MD correlated strongly with *f*_h_ *D*_h_ (*ρ* = 0.90, *p* = 4 × 10^−6^, Spearman), as predicted from Eq. 4. Time from onset, patient age, and *f*_r_ did not correlate significantly with any parameter. These correlations suggest that variations in the exchange rate may be responsible for determining the MD and the FA of stroke lesions in the sub-acute stage. Since MD increases and FA decreases from the early sub-acute stage onwards, a correlation between time from onset and *k* would have been expected, but it was absent. This absence could possibly be explained by the large heterogeneity in MD and FA observed between patients and within lesions [170, 171]. Follow-up of a cohort of patients with regular measurements could allow this hypothesis to be tested.

### Simulations of tissue undergoing ischaemia

Budde and Frank suggested that the total cell surface is preserved when cells swell during ischaemia, which would result in axon and dendrite beading [172]. Monte Carlo simulations of water diffusing in beaded axons showed that this is sufficient to explain a large decrease in AD, MD, and FA. The results were validated by subjecting excised rat sciatic nerve to stretching, which induces beading, but not a bulk shift of water into the axon. The beading mechanism could explain the simultaneous decrease in MD and FA between the hyper-acute and acute stage, but not the simultaneous decrease in FA and increase in MD during the sub-acute stage. However, the latter observation could possibly be explained by exchange as discussed above (Fig. 13).

Other explanations for the reduced MD in stroke have also been suggested based on simulation studies. For instance, Hall and Alexander investigated effects of tissue swelling on the diffusion weighted MRI signal using Monte Carlo simulations and noted that swelling may introduce regions of restricted diffusion in the extracellular space [106]. The authors suggested that this could explain the drastic MD reduction in stroke. In contrast, Jin et al. suggested that cell swelling results in the shrinkage of larger domains in the extracellular space rather than closing of the intercellular gap [107]. Harkins et al. [97] reproduced the large reduction in MD observed in stroke by simulating diffusion experiments in a two-compartment system. The large MD reduction was explained by the increase in intracellular volume fraction and by assuming that the *T*2 relaxation time is much shorter in the intracellular space than in the extracellular space. However, the MD would be highly dependent on TE under such conditions, in contrast to what has been observed experimentally [173].

### Altered intracellular diffusivity

Several authors have tried to perform separate investigations of the intra- and extracellular diffusivities in stroke lesions. For example, Silva et al. [174] measured the ADC in rats where the relaxivity was selectively enhanced in the extracellular space, and tuned the echo time so that only intracellular signal contributed to the measured ADC value. No major differences in the ADC values were observed between normal measurements and relaxation-enhanced measurements, indicating that there is no difference in ADC between the intracellular and extracellular space, or that the extracellular signal fraction is negligible. Following middle cerebral artery occlusion, the ADC was reduced by approximately 40 %, for both the normal and the relaxation-enhanced measurement. The reduction in *D*_intra_ following ischemia is also supported by reports of a reduced value of *D*_intra_ immediately after death, as observed in diffusion experiments with sub-millisecond diffusion times achieved by the use of oscillating gradients [155]. Doung et al. [175] determined the ADC of intracellular- and extracellular-specific molecular markers and did not detect any difference in ADC in the two spaces. Similarly, Neil et al. reported that the ADC of ^133^Cs that accumulated intracellularly was reduced in global brain ischaemia.

While these studies do suggest that *D*_intra_ is reduced following an ischaemic stroke, it is not clear what to expect regarding *D*_r_. Reduction of *D*_i_ leads to lower values of *α* and *β* (Eq. 6), which could actually result in increased values of *D*_r_ (Fig. 2). Separate measurements acquired with varying diffusion times would be required to better understand the implications of reduced values of *D*_intra_ on metrics observed by conventional diffusion MRI.

## Other applications

Conventional DTI has numerous clinical applications [176], and biophysical modelling of diffusion in white matter can help understand the mechanisms underlying alterations in DTI parameters. For example, Sen and Basser concluded that MD and FA are primarily influenced by changes in the outer diameter of axons, the extracellular volume fraction and the inter-axonal spacing [177]. Harkins et al. [97] used simulations to show that the ADC is nearly insensitive to variations in the membrane permeability. Nilsson et al. [80] suggested that stretching of nerves composed of undulating axons could increase the FA, based on results from simulations. Despite the progress made by such modelling studies, two shortcomings intrinsic in DTI remain: that the resulting parameters only indirectly related to the tissue microstructure [178], and that results can be confounded by the presence of crossing fibres and partial volume effects [179–181]. Due to such shortcomings, DTI results must be carefully scrutinized to avoid the misinterpretation that follows if FA interpreted is a measure of “white matter integrity” [182]. This claim is exemplified by the counterintuitive finding of elevated FA in a region of the brain of patients with mild cognitive impairment [183]. This result was interpreted as the relative sparing of motor-related pathways compared to cognitive-related ones in areas of crossing fibres, resulting in an increased homogeneity of fibre orientations.

To solve the problems intrinsic in DTI, biophysical models can be used to extract parameters more specific to the tissue microstructure, from data acquired with extended protocols. Such extended protocols may feature *b*-values higher than those used in DTI, which allows for crossing fibers to be resolved [179, 184]. Acquiring data with higher *b*-values may also increase the sensitivity to tissue microstructure alterations, which was explored in early studies of diseases such as multiple sclerosis [185], vascular dementia [186], and to follow up of treatment in intracranial tumours [187]. However, model-based assessment of microstructural properties such as the axon diameter or the intracellular exchange time require data to be acquired not only with higher *b*-values than in DTI, but also with variable diffusion times. Acquisition of such data comes with a price: longer scan times. This problem can be partially solved by improved pulse sequence design, as in the case of filter exchange imaging [87, 103], by using algorithmic protocol design [67], or by relevant simplifications of complex models as in the case of NODDI [82]. As many of the models and strategies described in this review have only recently been developed, their clinical applications are yet scarce, but that may change in the near future due to recent improvements in hardware and data acquisition strategies [188].

## Conclusions

Biophysical models of diffusion in white matter have been constructed to include effects of restricted diffusion in approximately cylindrical axons, a distribution of axon diameters, orientation dispersion and exchange between the intra-axonal and extra-axonal space, allowing these properties to be inferred from diffusion MRI experiments. In practise, however, properties such as parameters of the axon diameter distribution may be possible to estimate accurately in vivo only if limitations in the scanner hardware are overcome, most notably, limited values of *g*_max_. In general, the predictions made based on biophysical models agree with experiments performed in vivo. For example, the value of the signal fraction of slowly diffusing water agrees with the expected, assuming that myelin water is invisible at the long echo times at which diffusion MRI is performed. The specific tissue properties that determine the characteristics of the slowly diffusing water are, however, not yet fully characterized, although the water exchange rate and degree of orientation dispersion probably both contribute. Accordingly, those tissue properties also affect parameters determined using models such as DTI and DKI. For example, MD and FA are probably influenced by the degree of axonal undulation in extracranial nerves, while they correlate with the exchange rate in subacute stroke lesions. Recent studies have also shown that three-dimensional properties of white matter are required to take into account in order to further understand how the tissue properties affect the outcome of diffusion MRI experiments.

## Abbreviations

ADC: Apparent diffusion coefficient
MD: Mean diffusivity
FA: Fraction anisotropy

## List of symbols related to the diffusion MRI experiment

*g*, **n**, *δ*, ∆: Amplitude, direction and duration of diffusion encoding gradients, and the time between their leading edges
*t*_d_: Diffusion time
*b*, *q*: The magnitude of diffusion encoding, with *b* = (2π*q*)^2^·*t*_d_

## List of model symbols, used with subscripts *j*

*S*_j_: Intensity of diffusion encoded signal
*f*_j_: Signal fraction
*D*_j_: ADC of a component
RD_j_: Radial diffusivity, i.e. ADC in the direction perpendicular to a nerve
AD_j_: Axial diffusivity, i.e. ADC in the direction parallel with a nerve
RS_j_: Radial MRI signal intensity, i.e. *S* obtained with diffusion encoding in a direction perpendicular to the nerve

## Subscripts j

r/h: Restricted/hindered diffusion component
f/s: Fast/slow diffusion component

## Other model symbols

*d*: Axon diameter
*g*: Ratio between inner and outer axon diameter
*D*_intra_: Intracellular or intra-axonal diffusion coefficient
*D*_bulk_: Diffusion coefficient of the bulk medium
**u**, *θ*, *ψ*: Direction of the nerve, specified by polar and azimuthal angles
*α*, *β*: Symbols relating *δ* and ∆ to *d* and *D*_intra_
*k*, *τ*_i_: Exchange rate and intracellular exchange time
*P*_d_: Diffusional membrane permeability
*A*/*V*: Area to volume ratio
*λ*: Tortuosity of the extracellular space
*v*_extra_, *v*_axon_, *v*_myelin_: Volume fractions of the extracellular, intra-axonal and myelin spaces, respectively
*w*, *L*: Width of nodes of Ranvier, and internode length
